# The Edgar Speyer Prize Competition

**Published:** 1906-03-31

**Authors:** 


### HOSPITAL ADMINISTRATION.

### CONSTRUCTION AND ECONOMICS.

### THE EDGAR SPEYER PRIZE COMPETITION.

#### AN OUTSPOKEN CRITICISM.

I desire to avail myself of the invitation given in your last issue to hospital officials to express their views upon the subject of the recently published prize essays.

When it was announced last year by the King Edward's Hospital Fund, that Mr. Edgar Speyer had offered a prize of one hundred pounds for the best essay by a Hospital Secretary on "The economical management of an efficient voluntary Hospital," it was naturally expected that the offer of such a liberal reward would induce many of the more experienced hospital officials to enter the competition; when, however, the conditions attached to the offer were published, and it was found that they involved the publication of the name of the successful essayist, many who might otherwise have competed declined to do so. All who are practically conversant with the inner life of the hospital world, realise how necessary it is for a responsible paid official to be exceedingly circumspect in the public expression of his own opinions respecting the complex conditions of the work in which he is engaged, and upon the tactful discharge of which his position and livelihood depends. This consideration fully explains the reluctance which was manifested by many of the leading hospital officials to send in essays.

Mr. Speyer's Sole Object.

It may be taken for granted that the sole object which Mr. Speyer had in view in making his generous offer, was to secure an essay upon the best methods of hospital administration, which would prove of practical service to all hospital managers and responsible officials, and it was only a reasonable expectation that such an essay would be forthcoming. We are not informed to whom the difficult task of adjudicating upon the essays was entrusted, but we may assume that the judges were selected by Mr. Speyer on account of their practical knowledge of the details of hospital administration, and we can therefore only conclude that the rejected essays were not deemed equal in merit to the one for which the prize was ultimately awarded. As, however, an additional special prize of £50 has been given to Mr. E. W. Morris for his essay, which, we are informed, "the judges decided was nearly equal in merit to the prize essay" by Mr. G. H. Hamilton, we are enabled to form an independent opinion as to the relative merits of the two published essays.

Mr. Morris's Paper.

After a careful perusal of these essays, I have not the slightest hesitation in stating my opinion that Mr. Morris's paper is decidedly superior, both as regards its composition and practical value; indeed, I wish to express my warm appreciation of his very able and well-expressed essay, which, as far as it goes, is a really valuable contribution to hospital literature, and contains many suggestions and criticisms which are well worthy of careful consideration by all who are engaged in the management of our voluntary medical charities. I do not propose here to discuss Mr. Morris's paper in detail, as I desire to make some remarks respecting the prize essay; I may, however, point out, in passing, that Mr. Morris has very naturally devoted most attention to those aspects of hospital administration of which his personal experience has given him practical knowledge. His observations upon the buying and distribution of stores, more especially of those connected with the dispensary department, are thoroughly practical and ought to prove very helpful to his fellow workers. I can only express my regret that Mr. Morris did not see his way to deal more fully with several other important aspects of the subject.

The Prize Essay.

Turning now to the prize essay, I am sorry that I cannot express an equally high opinion of Mr. Hamilton's paper. I should have been glad to have been able to do so out of regard for the author; on the other hand, I do not think that it is desirable that the general public should be allowed to conclude that this prize essay fairly represents the average knowledge and literary ability of hospital secretaries. The general impression made upon my mind by the prize essay is that it contains comparatively little that is either helpful or informing. A large proportion of the statements made are so perfectly obvious, and many of them are so trivial, as to be entirely useless for any practical purpose; while some of the opinions expressed are quite impracticable or are open to very serious objections. In his opening passages Mr. Hamilton informs us that his remarks apply to "a hospital of medium size, say 200 to 300 beds"—namely, about the size of Westminster or St. Mary's Hospitals—and this standard must be always borne in mind when considering his various suggestions. He states that a "proper constitution" is necessary for a voluntary hospital, but he does not give us any idea of what in his opinion a proper constitution consists, he informs us, however, that "the management is generally first of all in the hands of the Governors." No reference is made to the method by which the Governors are enabled to exercise their powers, nor does he refer to the paramount importance of the constitution providing for the frequent meetings of the

Executive body, which, if efficiency is to be assured, must exercise a direct and constant supervision over the various departments of the hospital. Some rather vague remarks are made respecting the representation of the medical staff on the managing body, a very difficult question, upon which there is a considerable divergence of opinion among hospital authorities.

### Paid Officials in Complete Control Condemned.

We next come to the statement (on page 7) that "If it is possible to find a Chairman or a Treasurer who is able to devote constant attention to the work, it will not be necessary to have a paid official in complete control, when the Board is not sitting." This remarkable opinion is emphasised on the following page, where Mr. Hamilton adds the ambiguous suggestion that "to such a director will be referred any difficulty as to duties on the border line between two departments," while further on we are told that the whole of his scheme for duties of the various offices "has been drafted on the assumption that an honorary director is available."

I cannot believe that any experienced hospital manager can be found who will endorse Mr. Hamilton's proposal as either practicable or expedient; even a large endowed hospital like Guy's has found it advisable to supplement the post of Treasurer by the appointment of a paid official, to whom is entrusted the general superintendence of the hospital.

### The Secretary and his Duties.

When we come to his observations respecting the duties of the Secretary, we can only express our surprise that a person who has had practical experience of that position could hold such views. In the first place no mention whatever is made of a secretary's special functions, such as attending Committees, keeping minutes and accounts, conducting correspondence, and the numerous other duties, which must make the first demands upon his time and energies; while, on the other hand, we are favoured with a long and detailed list of "his main duties." most of which must of necessity, in a hospital of 200 to 300 beds, be deputed to other officers, acting under the Secretary's general supervision. It is practically impossible for any secretary, without neglecting his proper work, to personally direct the male servants, engineers, and workpeople, etc., including "the bathing of male patients."

### "Man" Versus "Woman."

I can only refer to the expression of opinion (on page 9) that "the actual reception and distribution of goods is generally more economically carried out by a woman than by a man," and his advice that "a housekeeper be appointed in preference to a steward." It is evident that both these officers are absolutely necessary in any efficiently managed hospital of the size indicated by the author. I anticipate that this and several other equally startling proposals will receive attention from other hospital officials who have practical experience of the work of the various departments referred to in the essay. I am afraid that I have already trespassed too much upon your valuable space, otherwise I would have referred to numerous other statements and opinions in the essay which seems to me to indicate a want of practical acquaintance with the economical management of an efficient voluntary hospital.

Yours faithfully,

March 26, 1906. "A Hospital Official."

